# Assessment and Predicting Factors of Repeated Brain Computed Tomography in Traumatic Brain Injury Patients for Risk-Stratified Care Management: A 5-Year Retrospective Study

**DOI:** 10.1155/2016/2737028

**Published:** 2016-09-15

**Authors:** Preeda Sumritpradit, Thitipong Setthalikhit, Sorayouth Chumnanvej

**Affiliations:** ^1^Acute Care Unit, Department of Surgery, Faculty of Medicine Ramathibodi Hospital, Mahidol University, Bangkok, Thailand; ^2^Neurosurgery Division, Department of Surgery, Faculty of Medicine Ramathibodi Hospital, Mahidol University, Bangkok, Thailand

## Abstract

*Background and Objective.* To determine the value of repeated brain CT in TBI cases for risk-stratified care management (RSCM) and to identify predicting factors which will change the neurosurgical management after repeated brain CTs.* Methods.* A 5-year retrospective study from January 2009 to August 2013 was conducted. The primary outcome was the value of repeated brain CT in TBI cases. The secondary outcome is to identify predicting factors which will change the neurosurgical management after repeated brain CTs.* Results*. There were 145 consecutive patients with TBI and repeated brain CT after initial abnormal brain CT. Forty-two percent of all cases (*N* = 61) revealed the progression of intracranial hemorrhage after repeated brain CT. In all 145 consecutive patients, 67.6% of cases (*N* = 98) were categorized as mild TBI. For mild head injury, 8.2% of cases (*N* = 8) had undergone neurosurgical management after repeated brain CT. Only 1 from 74 mild TBI patients with repeated brain CT had neurosurgical intervention. Clopidogrel and midline shift more than 2 mm on initial brain CT were significant predicting factors to indicate the neurosurgical management in mild TBI cases.* Conclusion*. Routine repeated brain CT for RSCM had no clinical benefit in mild TBI cases.

## 1. Introduction

Traumatic brain injury (TBI) patients with equivocal findings of brain computed tomography (CT) at the initial presentation frequently have repeated brain CTs. Repeated brain CT is commonly practiced at several trauma centers without protocol in place. Utilization of brain CT has increased over time; however, effects on outcome and associated risks are unknown. Brain CT may provide earlier identification for a type of traumatic brain injury. As a result, this patient will be receiving more aggressive neurosurgical intervention. In Ramathibodi Hospital, there is no protocol in place regarding repeated brain CT for TBI patients and it is controversy. So, based on physician preference and patient safety, repeated brain CT is still exercised. Regarding the patient safety, the risk-stratified care management (RSCM) is the interested procedure to assign a health risk status to a patient and to directly improve care management. Repeated brain CT is the option and the only one of the investigation of choice for the equivocal condition to establish TBI patient risk status as an objective tool. The apparent benefit of repeated brain CT was determined.

## 2. Material and Methods

A retrospective study of consecutive adult patients admitted to Acute Care Unit, Surgery Department, Faculty of Medicine Ramathibodi Hospital, after head injury was carried out after IRB approval. Data were collected from January 2009 to August 2013. Inclusion criteria were age more than fifteen years and duration of admission less than seventy-two hours. Patients who were treated with supportive treatment after initial brain CT were recruited. There were 145 cases potentially eligible. Variables collections including age, sex, underlying medical problems and medication, initial brain CT results, indication for repeated brain CT, Glasgow Coma Scale, Injury Severity Score, and treatment following repeated brain CT were determined.

### 2.1. Definition

Repeated brain CT was ordered by the neurosurgeons after personal assessment of TBI patient even though there were equivocal findings from initial brain CT and still no neurological deterioration. This is classified as repeated brain CT. Neurologic deterioration means alteration of neurologic status from neurological examination such as alteration of consciousness, limb weakness, lateralizing signs, and sudden appearance of severe symptoms such as headache, vomiting, and dizziness. Progressive hemorrhagic injury was identified when comparing repeated brain CT with initial brain CT. If there were any findings, even one or more of these findings will be included such as increasing in volume or size of hematoma/contusion, increasing of edema effect, or appearance of new lesions. The criteria for classification of TBI patients included mild TBI Glasgow Coma Scale (GCS) = 15–13, moderate TBI GCS = 9–12, and severe TBI GCS ≤ 8. Regarding the surgical intervention criteria for mild TBI cases, increasing of hematoma size more than 30%, increasing of hematoma volume ≥ 30 cc, surfacing location of hematoma from brain CT, asymmetrical basal cistern, and interval increasing of midline shift more than 2 mm are the indication for surgery after repeated CT head in mild TBI cases. All the measurement was determined by the software in PAC system. The sequential CT data sets were measured using the software, Volume Viewer Package on an Advantage Workstation 4.4 (GE Healthcare, Little Chalfont, UK) by neurosurgeons.

### 2.2. Statistical Analysis

Statistical analysis was done using StataCorp 2013, Stata: Statistical Software, College Station, TX: StataCorp LP, version 12. The *P* value < 0.05 is the level of statistical significance.

## 3. Results

**Figure 1:**
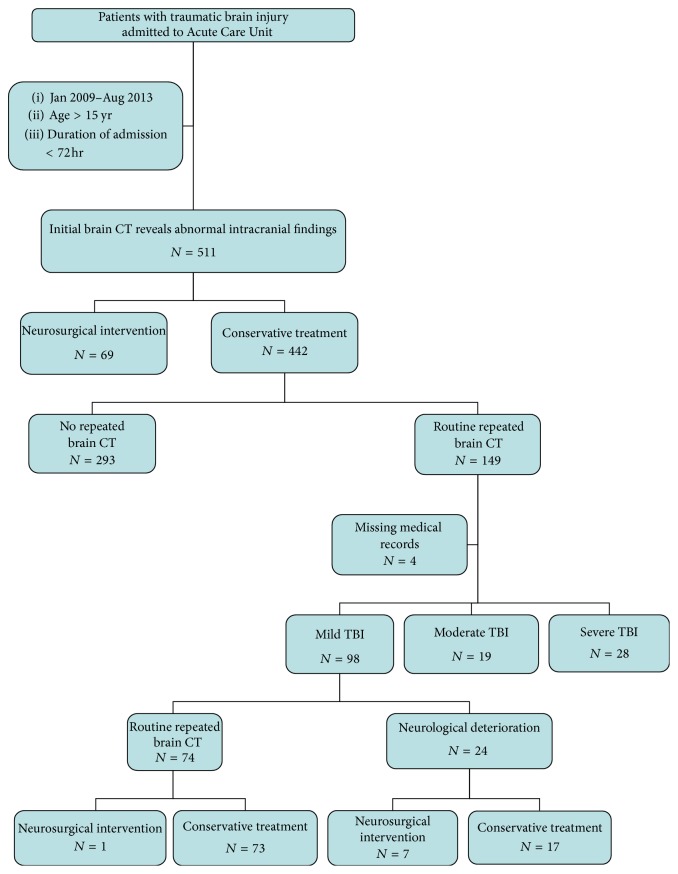
Study flow diagram.

There were 145 patients with traumatic brain injury and repeated brain CT potentially eligible. Around 67.6% of all cases (*N* = 98) were categorized as mild head injury, 13.1% of all cases (*N* = 19) as moderate head injury, and 19.3% of all cases (*N* = 28) as severe head injury (Figure 1). The mean (SD) age of the population was 52.0 (22.7) years (median age, 51 years; range, 15–93 years), with 71 of the 145 patients (48.9%) being younger than 50 years of age. About 69.7% of all cases (*N* = 101) were men, with a mean (SD) ISS of 20.6 (9.2) (median ISS, 20; range, 1–75), and 44.8% of all cases (*N* = 65) were in traffic accident from Table 1. In all 145 consecutive patients, 7.6% of cases (*N* = 11) had neurosurgical intervention. For mild head injury, 8.2% of patients (*N* = 8) underwent immediate surgery after repeated brain CT. 74 of 98 patients had been ordered for repeated brain CT investigation. Only 1 from 74 patients (1.35%) had neurosurgical intervention. And 24 of 98 patients had been ordered for emergency brain CT because of neurological deterioration. Seven of them (29.1%) had undergone operation. Comparing with conservative treatment patient group, old age, more underlying disease, and higher ISS and AIS were the conditions indicated for neurosurgical intervention. Clopidogrel (OR, 10.2 [95% CI, 1.87–55.38]) and midline shift more than 2 mm on initial brain CT (OR, 11.9 [95% CI, 2.50–57.20]) were statistical significance predicting factors for patients with mild head injury to undergo operation besides neurological deterioration in Tables 2-3. Indication for emergency CT head is based on alteration of consciousness, progressive weakness, and lateralizing signs and became confusing. Two of these patients denied surgery when neurosurgeon suggested with surgical indication and finally expired. Three patients underwent a craniotomy with clot removal; 2 patients underwent craniectomy. The remaining 1 patient underwent a burr-hole operation in Table 4. Only one patient after repeated brain CT had neurosurgical intervention who was 44-year-old female with motorcycle accident. Glasgow Coma Scale (GCS) at emergency unit was 13 and initial brain CT showed acute epidural hematoma 1.7 mm. After conservative treatment, GCS was still the same but after repeated brain CT there was a progression of hematoma size from 1.7 to 19 mm. Finally, this case was indicated for neurosurgical intervention. Sixty-one cases from 145 patients (42% of cases) revealed progression of intracranial hemorrhage, and 9 patients (15% of cases) needed neurosurgical intervention. Comparing repeated and initial brain CT, the mild TBI case was presented in Table 5 and the moderate or severe TBI was shown in Table 6. No statistical significance exists between severity of head injury and progression of hemorrhage (*P* value = 0.186). Of overall 98 cases of mild TBI, about 91.8% (*N* = 90) of cases were under conservative treatment. As a result, 8.2% (*N* = 8) of cases underwent neurosurgical operation.

## 4. Discussion

Brain CT is the investigation of choice to determine the severity and type of TBI cases. It is also the objective tool to evaluate the risk status for this patient group; however, to follow up duration with repeated brain CT is still controversial. Progressive hemorrhagic injury from repeated brain CT was reported about 32.3–43.6% [1–4] in spite of no neurological change in TBI cases. There were several studies stated about controversy in clinical valuable as a routine serial brain CT without neurological examination [5–12]. In this present study, the result revealed that less than 10% of mild TBI underwent neurosurgical intervention. In addition, trips to CT scanner may be associated with adverse events such as extubation or cardiac arrest [13]. And also routine repeated brain CT increases patient exposure to more radiation may increase risks of cancer [14]. Some previous studies have suggested that repeated brain CT is unnecessary in patients who remain neurologically stable [3, 5–7, 15, 16]. Others argue that repeated imaging is necessary because brain injuries can progress without neurologic changes [8–12]. However, a number of objective factors might help the physicians to make some decision for patient treatment plan or RSCM. Some interesting factors in this study including use of clopidogrel or brain CT revealing midline shift > 2 mm were the predicting factors for neurosurgical intervention indication. These 2 factors might be used to identify high risk patient for repeating brain CT. Regarding RSCM, the risk status is the main concern and should be an objective factor based on patient safety. Because of brain CT technology, it is an objective option to determine traumatic brain injury risk status. But, in this present study, the chance or clinical benefit to utilize repeated brain CT to determine progressive hemorrhagic injury in mild TBI patient is not compulsory to use as routine or without neurological examination as mentioned above. In mild TBI cases, brain CT should be realized that initial brain CT within 3 hours of injury is considered too early and recommended to repeated brain CT again within 12 hours [17, 18]. Otherwise, the physician would miss and could not detect progressive brain lesion. In moderate and severe TBI cases, CT scans are usually obtained within a few hours after injury. The subsequent scans in these groups will reveal different findings and clinical deterioration also plays a great role in the decision-making. These patients usually have worse outcomes regardless of whether surgery is performed [18]. In conclusion, the clinical recommendation for an interval of repeated brain CT should be 8–12 hours after injury [17]. In recent times, more elderly patients and antiplatelet or anticoagulation patients are the major concern. And also there is higher prevalence of chronic and multiple illnesses and too early brain CT investigation so one should be cautious of the missing or delayed progressive intracranial hematoma such as chronic subdural hemorrhage in aging patients. Because of this, repeated brain CT should be considered as a lesson learned and be your own custom case.

## 5. Conclusion

Routine repeated brain CT for RSCM had no clinical benefit in patient with mild traumatic brain injury without neurological examination. Clopidogrel and midline shift more than 2 mm are the clinical predicted factors to indicate neurosurgical intervention after repeated brain CT.

## Figures and Tables

**Table 1:** Demographic and clinical characteristics.

Characteristic	Total (*n* = 145)
Male	101 (69.7%)

Age (year)	52.0 ± 22
>50 years old	71 (49%)

Mechanism	
Motor vehicle accident	10 (6.9%)
Motorcycle accident	44 (30.3%)
High fall	13 (9.0%)
Low impact fall	55 (37.9%)
Body assault	11 (7.6%)
Others	12 (8.2%)

Underlying disease	
Diabetes mellitus	23 (15.9%)
Hypertension	47 (32.4%)
Ischemic heart disease	14 (9.7%)
Cerebrovascular disease	11 (7.6%)

Medication	
Aspirin	26 (17.9%)
Warfarin	6 (4.1%)
Clopidogrel	8 (5.5%)

ISS	20.6 ± 9
ISS > 19	73 (50.3%)

Brain AIS	3.9 ± 1
AIS > 4	33 (22.8%)

Result of initial brain CT	
Epidural hematoma	28 (19.3%)
Subdural hematoma	92 (63.4%)
Subarachnoid hemorrhage	81 (55.9%)
Hemorrhagic contusion	82 (56.6%)
Intraventricular hemorrhage	10 (6.9%)
Diffuse axonal injury	14 (9.7%)
Skull fracture	61 (42.1%)
Base of skull fracture	14 (9.7%)

**Table 2:** Clinical characteristics between conservative treatment group and neurosurgical treatment group after routine repeated brain CT in mild TBI (GCS 13–15).

Characteristic	Conservative treatment (*n* = 90)	Neurosurgical treatment (*n* = 8)	*P* value
Age, mean (SD), years	56.5 (23.0)	65.3 (17.7)	0.295
Age > 50 y, *N* (%)	54 (60.0)	6 (75.0)	0.478

Male sex, *N* (%)	55 (61.1)	6 (75.0)	0.706

Traffic injury, *N* (%)	33 (36.6)	1 (12.5)	0.256

Underlying disease, *N* (%)			
Diabetes mellitus	17 (18.8)	3 (37.5)	0.354
Hypertension	38 (42.2)	6 (75.0)	0.135
Ischemic heart disease	10 (11.1)	3 (37.5)	0.070
Cerebrovascular disease	9 (10.0)	1 (12.5)	0.592

Medication, *N* (%)			
Aspirin	21 (23.3)	2 (25.0)	1.000
Warfarin	4 (4.4)	0 (0.0)	1.000
Clopidogrel	5 (5.5)	3 (37.5)	0.017

ISS, mean (SD)	18.6 (8.6)	21.2 (4.4)	0.405
ISS > 19, *N* (%)	37 (41.1)	5 (62.5)	0.282

Brain AIS, mean (SD)	3.7 (1.1)	4.5 (0.5)	0.080
AIS > 4, *N* (%)	18 (20.0)	4 (50.0)	0.073

SBP on admission, mean (SD), mmHg	150.7 (31.7)	164.3 (23.2)	0.240

Heart rate on admission, mean (SD), beats/min	85.9 (16.2)	96.5 (24.2)	0.095

Result of the first brain CT, *N* (%)			
Epidural hematoma	16 (17.7)	1 (12.5)	1.000
Subdural hematoma	51 (56.6)	7 (87.5)	0.136
Subarachnoid hemorrhage	50 (55.5)	3 (37.5)	0.464
Hemorrhagic contusion	46 (51.1)	3 (37.5)	0.715
Intraventricular hemorrhage	6 (6.6)	0 (0.0)	1.000
Diffuse axonal injury	5 (5.5)	0 (0.0)	1.000
Skull fracture	30 (33.3)	3 (37.5)	1.000
Base of skull fracture	5 (5.5)	1 (12.5)	0.409

Midline shift, mean (SD), mm	0.67 (1.5)	4.25 (2.1)	
Midline shift, median, mm	0	3.5	0.0015
Midline shift > 2 mm, *N* (%)	11 (12.2)	5 (62.5)	0.003

Duration from injury to the first brain CT, median, hr	2.9	1.2	0.495

Duration from injury to routine repeated brain CT, median, hr	46.9	38.7	0.603

**Table 3:** Determination of odd ratio and  *P* value in neurosurgical treatment group in mild TBI.

Variables	Odd ratio	95% CI	*P* value
Subdural hematoma	5.3	0.63–45.33	0.136
Hypertension	4.1	0.78–21.46	0.135
AIS > 4	4.0	0.91–17.55	0.073
Ischemic heart disease	4.8	0.99–23.19	0.070
Clopidogrel	10.2	1.87–55.38	0.017
Midline shift > 2 mm	11.9	2.50–57.20	0.003
Emergency brain CT on neurological deterioration	30.0	3.46–280.83	<0.001

**Table 4:** Clinical characteristics of neurosurgical treatment group in mild TBI with second brain CT.

Sex	Age (years)	Mechanism of injury	GCS at initial admission	ISS	AIS	Initial brain CT	Indication for routine repeated brain CT	GCS during 2nd brain CT	Comparison of the brain CT result between first and second brain CTs	Intervention
M	81	Low fall	15	25	5	SDH	Progressive weakness	15	No change	Burr hole

M	78	Low fall	15	25	5	SDH	Worsening conscious	7	Worse	Craniectomy with clot removal

M	65	Low fall	15	25	5	SDH	Worsening conscious	8	Worse	Died after denial of surgery

M	55	High Fall	14	20	4	SDH, contusion, SAH, skull fracture	Progressive weakness Worsening conscious	11	Worse	Craniotomy with clot removal

M	80	Low fall	15	16	4	SDH, contusion, skull fracture	Worsening conscious	7	Worse	Bilateral frontal craniectomy

M	38	Low fall	15	26	5	SDH, SAH, contusion	More confusion	14	No change	Craniotomy with clot removal

F	44	Motorcycle	13	16	4	EDH, SAH, skull fracture	Routine	13	Worse	Craniotomy with clot removal

F	82	Low fall	14	17	4	SDH	Worsening conscious	6	Worse	Died after denial of surgery

**Table 5:** Comparison of the result of routine repeated brain CT versus initial brain CT in mild TBI.

Traumatic brain injury	Result after routine repeated brain CT			Total
	Improved	No change	Worsened	
Mild TBI	24	38	36	98

**Table 6:** Comparison of the result of routine repeated brain CT versus initial brain CT in moderate and severe TBI group.

Traumatic brain injury	Result after routine repeated brain CT			Total
	Improved	No change	Worsened	
Moderate TBI	6	5	8	19
Severe TBI	4	7	17	28

Total	10	12	25	47

## Additional Points

*Strengths and Limitations of This Study*

- This present study is the observational single-center, retrospective design so the recruited cases and clinical management are the confounding variables.
- Being a retrospective study, this may confine only in our clinical findings and management. Consequently, our discussion is not the clinical guideline or consensus for overall management of TBI patients.
- Some missing data most likely to occur in this present study and also the confounders might affect the difference of the factors.
- Regarding the definitions of neurological examination, we did not differentiate between different degrees of weakness or deficit.
- The risk-stratified care management is the process of management for a risk status evaluation to help patients achieve the best quality of care and to prevent acceleration to higher-risk categories and higher associated costs.
